# Paroxysmal dyskinesia associated with hyperthyroidism in 7 cats: a novel manifestation of a metabolic encephalopathy

**DOI:** 10.1093/jvimsj/aalaf007

**Published:** 2026-01-21

**Authors:** Javier Espinosa, Irene Espadas, Athina Karpozilou, Tamara Heredia, Alenka Hrovat, Juan Torre, Annette Wessmann, Aga Zoltowska, Charlotte Dye, Juan José Mínguez, Christoforos Posporis, Patricia Álvarez

**Affiliations:** Neurology and Neurosurgery Service, IVC Evidensia, Pride Veterinary Referrals, Derby, DE24 8HX, United Kingdom; Neurology and Neurosurgery Service, Veterios Referral Veterinary Hospital, 28022, Madrid, Spain; Neurology and Neurosurgery Service, IVC Evidensia Southern Counties Veterinary Specialists, Ringwood, BH24 3JW, United Kingdom; Neurology and Neurosurgery Service, Anicura Ars Veterinary Hospital, 08034, Barcelona, Spain; Oakwood Veterinary Referrals, Willows Veterinary Hospital, Hartford, CW8 1LP, United Kingdom; Medivet Veterinary Clinic Hucknall, Nottingham, NG15 7BY, United Kingdom; Neurology and Neurosurgery Service, IVC Evidensia, Tierklinik Hofheim, 65719, Hofheim, Germany; School of Veterinary Medicine and Science, University of Nottingham, Sutton Bonington Campus, Loughborough LE12 5RD, United Kingdom; Internal Medicine Service, IVC Evidensia, Pride Veterinary Referrals, Derby, DE24 8HX, United Kingdom; Neurology and Neurosurgery Service, IVC Evidensia, Pride Veterinary Referrals, Derby, DE24 8HX, United Kingdom; Neurology and Neurosurgery Service, IVC Evidensia, Pride Veterinary Referrals, Derby, DE24 8HX, United Kingdom; Neurology and Neurosurgery Service, IVC Evidensia, Pride Veterinary Referrals, Derby, DE24 8HX, United Kingdom

**Keywords:** feline, movement disorder, dystonia, dopamine, episodes, endocrinopathy

## Abstract

**Background:**

Paroxysmal dyskinesia associated with hyperthyroidism (HT) is a well-described disorder in humans and has not been previously reported in cats.

**Hypothesis/Objectives:**

Paroxysmal dyskinesia might develop as a consequence of HT in cats, with improvement or cessation of the episodes once normal thyroid function is restored.

**Animals:**

Seven client-owned cats.

**Methods:**

Multicenter retrospective observational study comprising cats with clinical signs consistent with the clinical phenotype of paroxysmal dyskinesia based on video recordings, and a concurrent diagnosis of HT. Follow-up information was obtained by contacting referring veterinarians and owners.

**Results:**

Seven cats were included. No signs of neurological disease were observed between the episodes except for plantigradism in one cat. Brain MRI studies were performed in 2 cats, revealing no intracranial abnormalities. In 1 cat, a 2-h video-assisted wireless electroencephalography confirmed the absence of epileptic activity. Clinical signs associated with HT were noted in all but 1 cat. After treatment for HT, complete remission of paroxysmal dyskinesia episodes was achieved in all cases, with euthyroid state confirmed in 6 cats.

**Conclusions and clinical importance:**

HT should be considered a differential diagnosis in cats with paroxysmal dyskinesia, even in the absence of other suggestive signs. Similar to the analogous disorder in humans, the prognosis appears excellent once appropriate treatment for HT is initiated.

## Introduction

Paroxysmal dyskinesia (PD) is an increasingly recognized and well-documented subtype of movement disorder in animals.^1^^,^^2^ In dogs, it was first reported over 50 years ago.^3^ During the past decade, several scientific publications have contributed to the understanding of this disorder and its pathophysiology,^4^ providing detailed clinical and etiological classifications.^5^^,^^6^ Additionally, selected terminology from human medicine applicable to animals has been described.^1^^,^^4^

Conversely, scientific information on PD in cats is scarce. To date, the only published report of PD in cats was in the Sphynx breed.^7^ A genetic origin was hypothesized given the lack of identifiable causes in an overrepresented breed. Paroxysmal dyskinesia remains an enigmatic condition in cats, despite verbal reports and anecdotal videos available online suggesting its occurrence without reaching the scientific literature.

Hyperthyroidism (HT) is a widely recognized disorder in cats with extensively characterized systemic signs.^8^^,^^9^ Specific signs of neurological disease attributable to this endocrinopathy are rare and include intracranial and extracranial manifestations, with abnormal behavior (agitation, aggression), generalized weakness, and tremors most commonly described. ^10–12^ Less frequent signs of neurological disease include reactive seizures with an incidence of 4.8% in hyperthyroid cats^13^ and cranial nerve polyneuropathy.^14^ Resolution or amelioration of these clinical signs usually occur after normalization of thyroid function.^9^^,^^12^^,^^14^ Rarely, severe signs of neurological disease such as marked obtundation or even coma can occur in the context of hyperthyroid storm.^15^ Despite these varied neurological manifestations, no direct association between PD and HT is described in cats. In contrast, the relationship between abnormal thyroid function and hyperkinetic or hypokinetic movement disorders is well-established in human medicine,^16^ with the earliest publications dating back to 1903.^17^ Despite this long time frame, the exact pathophysiology remains incompletely understood, but an altered metabolism of dopamine is consistently proposed.^16^

Among the hyperkinetic movement disorders associated with HT in humans, tremors and chorea (a sudden, irregular, and transient low-amplitude contraction of muscle groups, most prominently affecting distal muscles)^4^ are the most commonly reported, followed by dystonia among others.^16^ While the main cause of HT in humans is an autoimmune disorder known as Graves’ disease,^16^^,^^18^ a benign adenomatous disorder is the most common etiology in cats. Notably, a direct correlation has been established between serum thyroid hormone levels and the severity of episodes in humans.^16^^,^^19^ Moreover, PD can occur as the initial manifestation of an underlying subclinical or mild HT.^20^

The aim of this study was to present a cohort of cats with PD and concurrent HT. Here, we describe the clinical presentation, diagnostic findings, treatment, and outcomes in a group of cats exhibiting analogous features to those reported in humans.

## Materials and methods

This multicenter, retrospective observational case series, conducted across 5 veterinary referral hospitals in the United Kingdom and Spain, was approved by the University of Nottingham’s School of Veterinary Medicine and Science Committee for Animal Research and Ethics (CARE; 4238 181024).

Inclusion criteria consisted of: cats of any breed, age, sex, and neuter status with video-documented episodes consistent with the clinical phenotype of PD based on previous descriptions^1^^,^^2^^,^^5–7^ and concurrent diagnosis of HT based on supportive laboratory results, with or without other clinical signs associated with the endocrinopathy. In addition to serum thyroxine assay, the minimum required clinicopathological testing included routine hematology and serum biochemistry or an elemental-point-of-care (EPOC, Idexx) with measurements including metabolites, hematocrit, ionized calcium, blood gases, and acid/base assessment. HT was diagnosed based on total thyroxine (tT4) or equilibrium dialysis free T4 (fT4) elevation according to predefined reference range as follows: Idexx Catalyst tT4 10-60 nmol/L, VPG tT4 15-50 nmol/L, Zoetis VetScan-V tT4 19-62 nmol/L, Lab Services equilibrium dialysis fT4 10-50 pmol/L.

The medical records of client-owned cats diagnosed with PD and concurrent HT between February 2019 and March 2025 were reviewed. Given the rarity of PD in cats and the lack of consistent use of routine thyroid testing in this cohort, a prevalence study was not feasible. Cases were identified from the institution leading the study as well as through professional networks, without systematic review of institutional medical record databases.

Collected data included signalment, history, inter-episode neurological abnormalities, clinical and neurological examination performed by board-certified neurology and internal medicine specialists along with the results of performed investigations and follow-up information. Reported features of the episodes included description, frequency, duration, and potential triggers.

The videos of at least 1 episode for each case were reviewed by a minimum of 2 board-certified neurologists. Characterization of the episodes as PD adhered to the features and terminology previously established in veterinary medicine for both cats and dogs.^1^^,^^4^^,^^5^^,^^7^ This included dystonic postures, dystonic movements, or a combination of these occurring in the context of a normal mental status, without autonomic signs (salivation, urination, defecation, or vomiting) and with no behavioral or any other clinical abnormalities after cessation of the episode, regardless of its duration. The specific features evaluated included the body parts involved and the pattern of distribution, classified as focal, segmental, or generalized according to previous descriptions.^1^ Additionally, the course of the episode was characterized as either evolving (abnormal movements or postures extending to another body part while still affecting the previous one), or migrating/shifting (involvement of a new body part after resolution of the previously affected one).

Additional diagnostic data were collected when available: blood pressure measurements (with values ≥160 mmHg indicative of systemic hypertension),^21^ urinalysis (with reference values according to reported guidelines^22^ and urine protein/creatinine ratio >0.4 considered diagnostic of proteinuria),^23^ abdominal ultrasonography, thoracic radiographs, echocardiography, brain magnetic resonance imaging (MRI), cerebrospinal fluid (CSF) analysis with reference values according to reported range^24^ and electroencephalography (EEG). Follow-up information, including a repeat tT4 serum concentration after initiating HT treatment when available, was obtained from medical records and telephone communication with referring veterinarians or owners when necessary.

Cases were excluded if they lacked video recordings of the episodes, exhibited intracranial abnormalities on brain MRI, abnormal CSF analysis, or EEG consistent with epileptiform discharges, or if a minimum of 3-month follow-up period after initiation of HT treatment was not available.

## Results

### Animals

Seven cats met the inclusion criteria whereas 4 cats were excluded due to absence of video recording of the episodes or insufficient follow-up. Demographic, clinical, and laboratory data for the included cases are provided in Table S1. All but one was Domestic Short Hair (DSH). The median age at presentation was 120 months (range 74-158) and the median body weight was 3.6 kg (range 2.5-5). All cats were neutered, comprising 4 females and 3 males. All cats were appropriately vaccinated; however, retroviral status was confirmed to be negative only in the 2 cases for which results were available. Four cats lived exclusively indoors, while the remaining 3 had an indoor-outdoor lifestyle.

A preexisting comorbidity was only identified in one cat in which a moderate obstructive hypertrophic cardiomyopathy was diagnosed six months before the diagnosis of HT and for which the cat was treated with atenolol (0.6 mg/kg Q12h PO).

The primary presenting complaint for all cats was the occurrence of episodes consistent with PD. Six cats (6 out of 7) presented to the neurology service across four referral institutions, while the remaining cat was evaluated by the internal medicine service at a different institution. The management of the latter was carried out with the guidance of the neurology service from the hospital conducting the study. The median duration from episode onset to presentation across the population was 3 weeks (range 0-24).

None of the cats were diagnosed with HT before the onset of PD episodes. However, anamnesis and retrospective evaluation of medical records revealed signs consistent with HT in 6 out of 7 cats, with weight loss being the most prevalent sign (Table S1). No other signs of neurological disease in the inter-episode period were reported in the included cases, and the neurological examination was normal in all but one case, in which pelvic limb weakness and plantigradism were noted. This cat also had a history of chronic vomiting.

### Episode semiology

Video recordings of at least 1 episode were reviewed for all cases. Videos of 4 cases are provided (Videos S1-S4,) with two of these videos recorded during consultation by a board-certified neurologist.

In all cats, the episodes were consistent with the clinical phenotype of PD, characterized by dystonic postures and movements. No consistent triggers were noted or reported in any of the cases. Detailed information about the clinical features of the episodes in each case are provided in Table S2.

The median duration of the episodes was 3 min (range 1-35) and the frequency varied considerably among cases. One cat presented with a 24-h history of acute severe cluster episodes with at least 15 events, including those that occurred at home and during hospitalization. The cat with the lowest frequency had 3 episodes over a 6-month period before presentation and diagnosis of HT.

Common features observed in all cats during the episodes included impaired balance, stiffness, dystonic movements equally affecting thoracic and pelvic limbs, and dystonic postures, with varying degrees of trunk kyphosis or twisting. All cats exhibited slowness (bradykinesia)^4^ as a consistent and commonly observed feature during at least some phases of the episodes.

In 4 cats, a focal, segmental, and generalized pattern were observed throughout the episode. The majority of cats (6 out of 7) showed an evolving pattern throughout the episode. However, a shifting/migrating pattern was initially observed in 2 out of 7 cats before acquiring an evolving pattern. Other detected features were transient inability to walk (6 out of 7), and dystonia affecting the tail (6 out of 7) and neck (4 out of 7). Less commonly, dystonic head tremor (3 out of 7) and facial twitching (1 out of 7) were noted.

### Clinical examination, investigations, and diagnosis

Physical examination was normal in 3 out of 7 cats, while in the remaining 4 a low body condition score, palpable goiter, and heart murmur were noted. The neurological examination was normal in all but one cat which showed a plantigrade posture and gait. Further information is provided in Table S1.

Complete hematology and biochemistry were performed in 6 out of 7 cats, and in the remaining cat a venous EPOC analysis was carried out. Relevant laboratory abnormalities included: abnormal serum activity of liver derived enzymes (6 out of 6), low concentrations of serum creatinine (3 out of 6) and potassium (3 out of 6). In one cat, the abnormally low serum potassium concentration was the attributed cause for the plantigradism noted on clinical examination.

The cats were diagnosed with HT via total thyroxine (tT4) analysis in 6/7 cats (median value of 136.4 nmol/L; range 86–205.7 nmol/L; reference range: 10-60/15-50/19-62 nmol/L depending on the laboratory used), and fT4 in the remaining cat (>140 pmol/L; reference range: 10-50) which previously had a normal tT4 as shown in Table S1.

Two cats underwent an MRI study of the brain using a high-field magnet (1.5 Tesla), which showed no intracranial abnormalities. Findings related to the extra-cranial structures were noted in both cats: unilateral otitis media, medial retropharyngeal lymph node mild enlargement and a cystic right thyroid mass, compatible with thyroid adenoma in one case; and bilateral otitis media and regional submandibular and retropharyngeal mild lymphadenopathy in the other. Cerebrospinal fluid analysis was performed in one cat and abnormalities were not detected.

One cat presented with cluster episodes unresponsive to midazolam (0.3 mg/kg IV) and levetiracetam (30 mg/kg IV). A 2-h-EEG showed no electrographic support for epilepsy, including during several sudden head movements, likely representing awakening events (brief transitions from deeper sleep to lighter sleep or wakefulness, typically accompanied by noticeable changes in EEG), as shown in Video S5. After initiation of thiamazole (2.5 mg total dose Q12h PO), the cat acutely developed signs consistent with a hyperthyroid crisis (ventricular tachycardia, tachypnea, fever, diarrhea, and marked obtundation). Active cooling and an esmolol bolus (0.5 mg/kg IV), followed by constant rate infusion at a dose rate of 100 mcg/kg/min, lead to rapid stabilization of the clinical signs and cessation of the PD episodes within a few hours.

Additional investigations across this cohort included blood pressure measurements in 3 out of 7 cats, all of which were within normal limits. Urinalysis was performed in 4 out of 7 cats revealing proteinuria in three. Abdominal ultrasonography was conducted in 2 out of 7 cats, showing no abnormalities. Echocardiography was performed in 2 out of 7 cats, both of which exhibited hypertrophic cardiomyopathy phenotype at stage B1. Thoracic radiographs were taken in 3 out of 7 cats and no abnormalities were detected.

### Treatment and outcome

All cats were treated medically, six with thiamazole (2.5 mg total dose once or twice daily PO) and one with carbimazole (10 mg once daily PO). Three cats subsequently received radioactive iodine therapy (RIT). Treatment information for each case is shown in Table S2. One cat had an adverse reaction to thiamazole (2.5 mg total dose Q12h PO), including lymphadenopathy (reactive hyperplasia confirmed by fine-needle aspirates), facial pruritus, and vomiting. Consequently, oral treatment was discontinued leading to resolution of adverse reaction signs, and RIT was undertaken.

In the case with plantigradism, treatment with potassium (Kaminox; 0.5 mL BID Q12h PO for 6 weeks) was initiated 48 h before the referral consultation. Plantigradism resolved four weeks after potassium supplementation, coinciding with the normalization of serum potassium concentration. The same cat experienced resolution of chronic vomiting within two weeks of treatment for HT.

For all cats, the median follow-up was 8 months (range 3-12). A euthyroid state after initiation of HT treatment was confirmed in all but one case who received RIT and in which repeat tT4 measurement after treatment was not available. Full remission of PD episodes was reported in all cases, with complete cessation after treatment onset in 4 out of 7 cats and gradual reduction in frequency to final termination in the remaining cats after 2, 3, and 12 weeks as shown in Table S2.

## Discussion

This case series describes the simultaneous occurrence of PD and HT in cats, representing a potential novel sign of encephalopathy attributable to this endocrine disorder. This association is well established in humans, with an increasing number of scientific publications consistently reporting and validating its existence for over a century.^17^^,^^25–28^

Although brain MRI was performed in two cats, all cases included in the present study exhibited normal neurological examinations and no inter-episode abnormalities, supporting the absence of an underlying structural cause. One exception was a cat presenting with a plantigrade stance, suspected to be secondary to hypokalemia resulting from chronic vomiting associated with HT. The MRI studies revealed no intracranial abnormalities. Extracranial findings involving the tympanic bullae and regional lymph nodes were considered unrelated to the episodes of PD and most likely incidental, given the absence of specific clinical signs or progression without targeted treatment.

Paroxysmal dyskinesia is a subtype of movement disorder defined as an involuntary, sustained muscular contraction leading to transient abnormal postures, movements, gait, or a combination of these.^6^^,^^7^^,^^29–31^ This disorder can be classified based on various factors, including potential triggers^5^^,^^6^ or whether excessive or reduced movement predominates during an episode.^4^ As per the last review in dogs, the etiological classification of PD includes genetic, reactive (drug-induced, toxic, metabolic, or dietary such as gluten sensitivity), structural, and unknown causes.^5^

While a direct correlation between PD and gluten sensitivity is reported in dogs, particularly but not exclusively in the Border Terrier breed,^32^^,^^33^ no scientific data are currently available regarding a potential association between gluten sensitivity and PD in cats. In human medicine, associations between celiac disease and HT secondary to Graves’ disease are described^34^; however, this occurs within the context of a broader autoimmune process, which differs from the primary pathophysiology of HT in cats. No correlation with gluten sensitivity was suspected or investigated in this cohort, which was supported by the positive response to HT treatment without any dietary modifications after diagnosis.

The current knowledge of PD in cats is limited. The only scientific publication to date reports PD in a group of Sphynx cats for which a genetic origin was suspected based on the absence of abnormalities after investigations.^7^ No obvious triggers were reported in 7 out of 10 cats, and spontaneous remission occurred in the minority, 2 out of 10 cases. Notably, their younger age at onset (less than 4 years) contrasts with our cohort, where the median age was 10 years old, warranting further investigation into a potential acquired cause. Despite the seemingly different etiologies, the episodes observed in the cohort of Sphynx cats closely resembled the clinical presentation of the seven cats reported here, which was primarily characterized by dystonic postures and movements. In both groups of cats, the dystonic movements were mostly, but not exclusively, accompanied by slowness or bradykinesia, which is not a common observation previously described in dogs with PD.^4^^,^^5^

In humans, HT can affect the nervous system, causing signs with both central and peripheral components, including myopathies, neuropathies, and psychiatric disorders among other signs of encephalopathy. For instance, tremor is a very common clinical sign in humans with HT, affecting up to 76% of patients and sometimes considered a feature of HT rather than a neurological complication.^16^^,^^20^ Signs of neurological disease occur in cats with HT. One review specifically compared the neurological manifestations of HT in humans and cats showing similarities between these species such as weakness and behavioral changes.^12^ In humans, tremors and other signs of neurological disease such as PD might sometimes be the first indication of increased thyroid activity.^16^^,^^20^ Although HT has also been associated with tremors in cats (albeit with no robust data regarding prevalence), it has not yet been directly linked to PD.^9^^,^^12^ This contrasts with the rare but well-established association between HT and PD in humans,^16^ where chorea is the predominant subtype. Ballism, choreoathetosis, and dystonia are more rarely described,^16^^,^^18^^,^^25–28^ the latter being the main clinical sign in our cohort of cats. There is currently no available data on the overall prevalence of PD in humans with HT. Among subtypes, chorea has a prevalence of 2%. However, due to possible misidentification as severe restlessness, this is believed to be an underestimation.^16^

While the exact pathophysiological mechanism linking HT and PD in humans remains unknown, the catecholamine neurotransmitter dopamine^35^ is thought to play a central role. Dopamine receptors are located within the basal nuclei, which are intrinsically involved in the development of PD.^16^ A study from several decades ago demonstrated increased sensitivity of dopamine striatal receptors in hyperthyroid guinea pigs.^36^ Furthermore, lower concentrations of homovanillic acid (a major catabolite of dopamine)^37^ was found in humans with HT, suggesting a reduced turnover.^38^ Lastly, a dopamine antagonist such as haloperidol has reported efficacy in the treatment of hyperthyroid-related chorea.^20^^,^^39^ In cats, while a similar pathophysiology might be suspected, it remains a hypothesis.

In people, paroxysmal dyskinesia usually occurs in a context of clinically evident HT. Nevertheless, PD can also develop as the first sign of subclinical HT, as in one of the cats reported here^16^^,^^27^^,^^28^^,^^40^ in which no clinical signs suggestive of HT were noted before the onset of PD despite tT4 being markedly elevated, likely representing an incipient or subclinical form of HT. In people, it is also accepted that PD could represent a sign of an already treated yet poorly controlled HT.^16^ However, none of the cats presented here were diagnosed with HT or received specific treatment for HT before the onset of PD episodes. Thus, as in humans, we propose that PD might serve as an early indicator of HT in cats, and that measurement of tT4 should be considered in middle-aged cats presenting with PD.

Another cat had normal tT4 levels, yet markedly elevated fT4. Up to 10% of hyperthyroid cats, and 30% with incipient or subclinical HT might have normal tT4,^41^^,^^42^ known as occult HT. This highlights the relevance of fT4 when HT is suspected despite normal tT4.

In humans, there is also documented direct correlation between the intensity of PD episodes and serum thyroid hormone levels.^19^ In this case series, the cat with the highest tT4 concentration had the most severe presentation with multiple cluster episodes and concurrent hyperthyroid crisis, during which there is an increased sensitivity to catecholamines with upregulation of β-adrenergic receptors. This leads to an enhanced sympathetic activity in the presence of diminished parasympathetic tone,^9^^,^^43–45^ which explains, at least partially, the beneficial effects of β-blockers (ie, esmolol) in ameliorating hyperthyroid-related clinical signs^46^^,^^47^ (tachycardia, breathlessness, restlessness, irritability, tremors) including cluster episodes of PD in this cat. However, no other clear association between tT4 concentration and the intensity, duration, or frequency of PD episodes was observed in the rest of the cases.

Another relevant feature of PD associated with HT in humans is a parallel temporal relationship between PD remission and attainment of euthyroid state.^18^^,^^40^ The frequency of PD episodes subsides within weeks of initiation of HT treatment followed by complete cessation of episodes once a sustained euthyroid state is reached.^16^^,^^20^^,^^27^^,^^28^ This correlates with the findings reported here, with euthyroidism confirmed in 6 out of 7 cats. Repeat tT4 analysis was not available at follow-up for the remaining cat, yet the resolution of HT related clinical signs together with the cessation of the PD episodes, highly supports its relationship. Although infrequent, persistent, long-term episodes of PD might persevere in humans despite normalization of thyroid function,^16^^,^^48^^,^^49^ but this was not observed in our cohort of cats in the included follow-up.

The main limitations of this study are the small number of cases and its retrospective design, which impaired homogeneity of diagnostic tests and treatment protocols across the population of cats. Magnetic resonance imaging study of the head was available in only two cases, which limits the ability to fully rule out other structural intracranial conditions. However, the fact that all included cases exhibited normal neurological examination both between episodes and throughout follow-up, along with complete resolution of clinical signs after specific treatment of HT and the absence of any other progressive deficits, make alternative diagnoses unlikely. Electroencephalography was only available in one case; and hence the diagnosis of PD was strictly made on phenotypic features in most of the cases. This approach, however, aligns with the common veterinary practice, as EEG is rarely feasible in clinical settings. The absence of epileptic activity on the EEG recorded within 24 h of multiple cluster episodes, combined with the consistent phenotype of these episodes in comparison with those of the other 6 cats in the study, support the diagnosis of PD. Nonetheless, the lack of EEG for all included cases remains a limitation. Another limitation is that gluten sensitivity was not investigated in our cohort of cats. A potential link between PD and gluten sensitivity, with or without concurrent HT, represents an open door for future research in cats. Lastly, confirmation of post-treatment euthyroid state in one cat that received RIT was not available. However, RIT is associated with a high success rate, with only 5%-10% of cats remaining hyperthyroid thereafter.^50^ This, combined with the resolution of the clinical signs attributable to HT in addition to those of PD supports the successful treatment and likely normalization of thyroid function.

In conclusion, this case series documents PD in association with HT in cats, that bears similarities to the analogous condition in human patients. HT should be considered as a differential diagnosis in cats presenting with PD, particularly in mature to senior cats in which the endocrinopathy is more prevalent, even in the absence of other suggestive signs of HT. While uncommon, a normal tT4 does not exclude HT, particularly when suggestive clinical signs, including PD, are present. The prognosis appears to be excellent when normal thyroid function is restored with medical therapy, RIT, or a combination of these treatment modalities, without the need for additional medications to specifically address PD.

## Supplementary Material

aalaf007_Supplemental_Files

## Off-label antimicrobial declaration

The authors declare no off-label use of antimicrobials.

## Institutional animal care and use committee or other approval declaration

Approval granted by the University of Nottingham’s School of Veterinary Medicine and Science Committee for Animal Research and Ethics (CARE) (4238 181024).

## Abbreviations

CSF: cerebrospinal fluid
DSH: domestic short hair
EEG: electroencephalography
fT4: free thyroxine
HT: hyperthyroidism
PD: paroxysmal dyskinesia
MRI: magnetic resonance imaging
RIT: radioactive iodine therapy
tT4: total thyroxine
