# Supplementary material for: Paroxysmal dyskinesia associated with hyperthyroidism in 7 cats: a novel manifestation of a metabolic encephalopathy
Source: J Vet Intern Med. 2026 Jan 21;40(1):aalaf007. doi: 10.1093/jvimsj/aalaf007 (PMC12881977; doi:10.1093/jvimsj/aalaf007)
Supplement: aalaf007_Supplemental_Files [file aalaf007_supplemental_files.zip › Supplemental_Table_1_aalaf007.pdf]

Supplemental Table 1: Demographic, clinical and laboratory data of cats with PD and HT

Case	Breed	Age	Sex	Weight (kg)	Time from onset to presentation	Previous signs of HT	Physical examination	Neurological examination	Haematology	Biochemistry	tT4
**1**	DSH	8y3m	FN	3.0	24 hours	Weight loss<br>Polydipsia	HM IV/VI<br>BCS 3/9	Normal (*)	Normal	K: 3.2 mmol/L (3.8-5.3)<br>CK: 1920 U/L (0-118)<br>AST: 82 U/L (0-26)	**205.7** nmol/L (15-50)
**2**	DSH	9y9m	MN	3.4	4 weeks	Weight loss<br>Polydipsia	Unremarkable<br>BCS: 4/9	Normal (*)	Normal	CREA: 75.0 umol/L (80-203)<br>UREA: 10.1 mmol/L (2.5-9.9)<br>ALT: 102.8 U/L (5-60)<br>ALP: 84 U/L (<60)	**162.5** nmol/L (10-60)
**3**	DSH	12y1m	MN	3.8	10 weeks	Weight loss<br>Restlessness<br>Polyphagia	Tachycardia<br>Goitre<br>BCS: 4/9	Normal	Normal	ALP: 195 U/L (10-90)<br>AMY: 1185 U/L (300-1100)<br>K: 3.4 mmol/L (3.7-5.8)	30 nmol/L (19-62)<br>EDfT4*: **140** pmol/L (10-50)
**4**	DSH	10y	FN	5.0	3 weeks	None	Unremarkable<br>BCS: 7.5/9	Normal	Normal	ALT: 361.8 U/L (10-85)	**136.42** nmol/L (10-60)
**5**	DSH	6y2m	MN	4.7	6 months	Stress-induced open-mouth breathing	HM III/VI<br>BCS: 6/9	Normal	Not performed<br>EPOC→ Haematocrit normal, pH: 7.061 (7.28-7.46), pCO2: 9.28mmHg (3.30-5.60), pO2: 24.2mmHg (12-14.7)	Not performed<br>EPOC→ Urea, creatinine, lactate and electrolytes normal	**103** nmol/L (10-60)
**6**	Manx	13y2m	FN	2.5	2 weeks	Weight loss<br>Polyphagia	HM: IV/VI<br>Tachycardia<br>Tachypnoea<br>BCS: 4/9	Normal	NEU: 17.39 x10^9/L (2.5-12.5)	P: 1.0 mmol/L (1.2 -2.6)<br>CREA 68 umol/L (80 -180)<br>ALP: 128 U/L (0 -50)<br>ALT: 78 U/L (0 -60)<br>CK: 260 U/L (0 -152)	**86.0** nmol/L (15-50)
**7**	DSH	13y	FN	3.2	3 weeks	Chronic vomiting	Unremarkable<br>BCS: 5/9	Plantigradism	Normal	ALT: 138 U/L (12-130)<br>CREA: 61.89 umol/L (80-203)<br>GLOB: 5.3 g/dL (2.8-5.1)<br>K: 2.7 mmol/L (3.5-5.8)	**154.26** nmol/L (15-50)

ALP: alkaline phosphatase, **ALT**: alanine aminotransferase, **AMY**: amylase, **AST**: aspartate transaminase, **BCS**: body condition score, **CK**: creatine kinase, **CREA**: creatinine, **DSH**: domestic short hair, **EDfT4**: equilibrium dialysis free thyroxine, **EPOC**: elemental-point-of-care, **FN**: female neutered, **GLOB**: globulins, **HM**: heart murmur, **HT**: hyperthyroidism, **K**: potassium, **MN**: male neutered, **NEU**: neutrophils, **P**: phosphate, **pCO2**: partial pressure of carbon dioxide, **PD**: paroxysmal dyskinesia, **pH**: potential of hydrogen, **pO2**: partial pressure of oxygen, **tT4**: total thyroxine. (*) One PD episode witnessed during examination
