# Supplementary material for: Paroxysmal dyskinesia associated with hyperthyroidism in 7 cats: a novel manifestation of a metabolic encephalopathy
Source: J Vet Intern Med. 2026 Jan 21;40(1):aalaf007. doi: 10.1093/jvimsj/aalaf007 (PMC12881977; doi:10.1093/jvimsj/aalaf007)
Supplement: aalaf007_Supplemental_Files [file aalaf007_supplemental_files.zip › Supplemental_Table_2_aalaf007.pdf]

Supplemental Table 2: Episode features, HT treatment and follow-up in cats with paroxysmal dyskinesia.

Case number	Episode description	Episode semiology	Episode duration (minutes)	Episode frequency	Treatment	Confirmed euthyroid state after treatment	Response	Time from onset of treatment to episode remission	Time of last follow-up since diagnosis
1	Dystonic posture (kyphosis) and movements (LT followed by RT), head tremor, tail rigidity, uncoordinated gait and facial twitching	Migrating followed by evolving pattern. Focal, segmental and generalised components	3	Cluster episodes over 24 hours	Carbimazole (10mg SID) Atenolol for 2 weeks (6.25mg SID)	Yes	Full remission	Immediate*	6 months
2	Dystonic posture (twisted trunk) and movement (PL evolving to LT), tail rigidity and stiff gait	Evolving pattern. Focal, segmental and generalised components	2	8 episodes in 4 weeks	Thiamazole (2.5mg BID) + RIT	Yes	Full remission	Immediate*	12 months
3	Dystonic posture (twisted trunk and flexed LP) and movements (RT), head tremor	Evolving pattern. Focal, segmental and generalised components	5-20	2 episodes in 10 weeks	Thiamazole (2.5mg BID) + RIT	NA	Full remission	Immediate*	8 months
4	Dystonic posture (trunk and neck) and movements (PL), tail rigidity and uncoordinated gait	Evolving pattern. Segmental and generalised components	2-4	7 episodes in 3 weeks	Thiamazole (2.5mg SID)	Yes	Full remission	2 weeks (1 episode between diagnosis and remission)	10 months
5	Dystonic posture (twisted trunk) and movements (RP and RT, evolving to LT and LP), stiff neck, head tremor, tail rigidity and uncoordinated gait	Migrating followed by evolving pattern. Focal, segmental and generalised components	1-5	3 episodes in 6 months	Thiamazole (2.5mg SID) + RIT	Yes	Full remission	3 weeks (4 episodes between diagnosis and remission)	12 months

6	Dystonic posture (kyphosis) and movements (TL), tail rigidity and stiff gait	Evolving pattern. Focal and segmental components	2-3	5 episodes in 2 weeks	Thiamazole (2.5mg BID)	Yes	Full remission	Immediate	3 months
7	Dystonic posture (kyphosis), body swaying, tail rigidity and uncoordinated gait	Generalised component.	10-35	5-10 episodes per day	Thiamazole (2.5mg BID)	Yes	Full remission	12 weeks (gradual reduction in frequency)	5 months

BID: bis in die (twice daily), **HT**: hyperthyroidism, **LP**: left pelvic limb, **LT**: left thoracic limb, **NA**: not available, **PL**: pelvic limbs, **RIT:** radioactive iodine therapy, **RP**: right pelvic limb, **RT**: right thoracic limb, **SID:** semel in die (once daily), **TL**: thoracic limbs *: No further episodes observed since the initiation of treatment
